# Prompting the Question: Evaluating ChatGPT-Generated Gross Anatomy and Embryology Questions for Accuracy and NBME (National Board of Medical Examiners) Item-Writing Style

**DOI:** 10.7759/cureus.107397

**Published:** 2026-04-20

**Authors:** Sofia Lopiano, Evan S Cohen, George Holan, Jeremy J Grachan

**Affiliations:** 1 Office of Education, Rutgers University New Jersey Medical School, Newark, USA; 2 Department of Medicine, Rutgers University New Jersey Medical School, Newark, USA; 3 Department of Surgery, Rutgers University New Jersey Medical School, Newark, USA

**Keywords:** anatomy education, assessment, chatgpt, medical education, national board of medical examiners (nbme), united states medical licensing exam (usmle)

## Abstract

Purpose

Current research has shown that Generative AI language models (e.g., ChatGPT) can answer and provide explanations for practice questions and generate new questions. Further investigation is needed to assess how ChatGPT-generated questions compare with National Board of Medical Examiners (NBME)-style questions to determine their quality.

Method

ChatGPT 4.0 was used to generate five multiple-choice questions using different prompts without learning objectives for gross anatomy and embryology content related to six organ systems between 2024 and 2025. An initial 10 prompts generated 300 questions, followed by 210 questions using seven modified prompts. Questions were evaluated based on 15 parameters reflective of NBME standards.

Results

Across the 10 initial prompts evaluated, 80% (n=240) of questions contained accurate information. Questions were classified as “higher order” 7.7% (n=23) of the time. Repeated words appearing in both the question stem and answer choice were present in 15% (n=45) of the questions. As the analysis progressed, prompts were continually modified to minimize lower-order questions and include more detailed vignettes. Using the modified prompts, the proportion of accurate questions decreased to 70.5% (n=148), but the proportion of higher-order questions increased to 41.0% (n=86).

Conclusions

ChatGPT tends to generate lower-order questions that assess a medical student’s foundational knowledge, rather than creating higher-order application and synthesis questions. However, the quality of the questions based on NBME item-writing standards is variable and still requires additional review before use. ChatGPT has the potential to increase faculty efficiency by streamlining question writing and serving as a supplemental study resource for medical students.

## Introduction

Medical students increasingly turn to ChatGPT to generate study materials and practice questions for self-study without providing source material [1]. Despite its prevalence, the practice of using artificial intelligence (AI) to generate practice multiple-choice (MCQs) questions without uploaded content is comparatively understudied. As generative AI models have demonstrated potential across a range of educational applications, including simulating clinical scenarios and patient interactions, supporting active learning modalities, interpreting anatomical images, and producing assessment content [2-10], their integration into medical education has accelerated. While many students are open to integrating ChatGPT into their learning, ways to implement the technology effectively remain an active area of investigation [11]. Yet, as AI-generated materials are increasingly incorporated into learning and assessment environments, concerns regarding their accuracy, cognitive rigor, and educational validity have emerged [12].

These concerns are particularly consequential in medical education, where assessment quality directly impacts student preparation for board examinations. The National Board of Medical Examiners (NBME) Item-Writing Guide establishes the standard for medical multiple-choice question construction, emphasizing clinical reasoning, avoidance of cueing, and well-structured vignettes, which are reflected across the Step examinations that United States-based allopathic medical students must pass [13]. Whether ChatGPT can reliably generate questions by meeting these criteria remains unclear.

Existing literature evaluating large-language model (LLM)-generated MCQs demonstrates variable performance. LLMs have shown the ability to achieve passing-level performance on standardized examinations and faculty-generated assessments [14-16]; yet, inaccuracies and inconsistencies in answer choice explanations persist [17,18]. Studies specifically examining the quality of ChatGPT-generated MCQs have similarly yielded mixed results, with several identifying a range of issues, including inaccuracy, vagueness, and misleading content in 12-25% of generated questions [19,20]. Although one study found ChatGPT-generated MCQs comparable to human-written questions in clarity and quality, they were deemed less relevant [21]. When comparing student performance on human versus ChatGPT-generated MCQs, one study found that while question difficulty was similar, human-written questions demonstrated greater discriminatory power in differentiating between high- and low-performing students, and suggested that prompt phrasing may influence ChatGPT's ability to produce questions with higher discriminatory value [22].

Despite this growing body of research, two critical gaps remain. First, most studies evaluating ChatGPT's ability to generate questions have used uploaded source material such as textbook chapters or self-learning guides [19-21], whereas its ability to generate questions without such input, a method most commonly used by students studying independently, is less studied. Second, while AI performance on anatomy and embryology questions has been explored [23], little research exists evaluating AI-generated questions against a standardized NBME-aligned rubric for the pre-clinical curriculum. While rubric-based evaluation of AI output has been explored in anatomy education in other contexts [9], its application to AI-generated MCQ quality assessment in the pre-clinical curriculum remains limited. High-quality medical exam questions aim to assess higher-order cognitive skills, moving beyond simple recall to application and analysis of clinical concepts, and it remains unknown whether ChatGPT can consistently meet this standard. This study addresses both gaps by assessing the accuracy and question quality of ChatGPT-generated gross anatomy and embryology MCQs for pre-clerkship medical students across 17 different prompts, without providing specific curricular content or learning objectives.

This article was previously presented as a meeting abstract at the 2025 NEGEA/NEGSA/NEOSR Joint Conference at the Hyatt Regency, New Brunswick, NJ, on April 2, 2025.

## Materials and methods

This study was conducted by the Office of Education at Rutgers New Jersey Medical School (NJMS) in Newark, NJ. The study utilized 17 different prompts in ChatGPT 4.0 to evaluate gross anatomy and embryology multiple-choice questions related to a specific body system that adheres to the NBME Item-Writing Guide [13]. Six organ systems were evaluated in this study: musculoskeletal, digestive, nervous, cardiovascular, respiratory, and urinary. These systems were chosen based on common topic areas that NJMS students ask for additional practice questions. Ten initial prompts were entered into ChatGPT to pilot the investigation. A separate browser was used for each prompt rather than a continual thread. Each prompt used was asked to generate five MCQs. The initial prompts were as follows: (1) "Create 5 multiple choice questions about the gross anatomy and embryology of the ____________ system." (2) "Create 5 multiple choice questions for medical students about the gross anatomy and embryology of the ____________ system." (3) "Create 5 USMLE style multiple choice questions about the gross anatomy and embryology of the ____________ system." (4) "Create 5 USMLE Step 1 style multiple choice questions about the gross anatomy and embryology of the ____________ system." (5) "Create 5 NBME style multiple choice questions about the gross anatomy and embryology of the ____________ system." (6) "Create 5 NBME Step 1 style multiple choice questions about the gross anatomy and embryology of the ____________ system." (7) "Create 5 multiple choice questions about the gross anatomy and embryology of the ____________ system following the NBME Item Writing Guide." (8) "Create 5 multiple choice questions about the gross anatomy and embryology of the ____________ system using clinical vignettes and following the NBME Item Writing Guide." (9) "Create 5 multiple choice questions using clinical vignettes to apply gross anatomy and embryology of the ____________ system to clinical scenarios and following the NBME Item Writing Guide." (10) "Create 5 multiple choice questions using clinical vignettes to apply gross anatomy and embryology USMLE learning objectives of the ____________ system to clinical scenarios and following the NBME Item Writing Guide."

Each question that ChatGPT produced was evaluated against 15 parameters, summarized into five criteria. These criteria were (1) accuracy, (2) whether the questions related to either embryology or gross anatomy concepts as defined by the topic of the question stem and its associated answer options, (3) the order level of the question based upon the Blooming Anatomy Tool [24], (4) grammatical accuracy, and (5) the presence of common item features and flaws as outlined in the NBME Item-Writing Guide [13]. The collective criteria and whether they should be present were combined into a rubric (Table 1). Descriptions of each criterion were reviewed through the respective sources [13,24] individually and collectively discussed as a research team before analysis of any MCQs.

**Table 1:** Criteria used to evaluate each question and its answers. ^a^Based on the Blooming Anatomy Tool [24] Other criteria are based on the National Board of Medical Examiners (NBME) Item-Writing Guide [13]

Criteria	Should Be Present?	Explanation
Overall		
Accuracy	Yes	Information presented in question stem is appropriate and correct; Single best option that correctly answers the question
Grammatically correct	Yes	No grammatical issues in the question stem and answer options
Question Stem		
Taxonomy^a^	Higher order	A 3-day-old infant is found to have a scaphoid abdomen and diminished breath sounds on the left side. A chest X-ray reveals abdominal contents in the thoracic cavity. Which of the following embryonic events likely led to this condition?
Includes a Vignette	Yes	
Negatives present	No	“Which of the following is not true about this patient’s condition?”
“Except” present	No	“Each of the following statements are true except:”
Complicated	No	Stems that include excess information that is irrelevant to answering the question and is put in the question stem to overwhelm the test-taker
Grammatical clues	No	“This patient would most likely be diagnosed with an:”, but then having only some options begin with a vowel; “Which of the following are”, but having only some options be plural
Answer Options		
Similar options	Yes	All options are from a related category (e.g., diseases, medications, anatomical structures”)
Complex	No	Very long, detailed options; overlapping numerical ranges (e.g., one option is Weeks 3-8, another is Week 5)
Similar length	Yes	All answer options are similar in length
Repeated words from stem present (i.e., “clang clues”)	No	Mentioning words or phrases in the question stem and in the answer options (e.g., the questions asking “Which of the following muscles, innervated by the ulnar nerve, is primarily responsible for flexing the digits?” and then having some options that mention “flexor” and/or “digits”)
None/all of the above present	No	Using “All of the above” or “None of the above” as answer options
Absolute Terms	No	Using “always” or “never”
Convergence	No	When the correct answer has similarities to other options, for example: A) Coracoacromial ligament B) Coracoclavicular ligament C) Glenohumeral ligaments D) Rotator cuff tendons E) Scapular spine Two options have “coraco-“, three options have “ligament”

The accuracy and concept were determined by two full-time medical school anatomy faculty members (authors GH and JG), who reviewed the questions and answer options individually, without knowing which prompt the questions were generated from, to minimize bias in the assessment of the questions. Discrepancies in question accuracy evaluation were found in only 20 of the 510 questions (percent agreement for the total 510 questions = 96.08%). For the 3.92% where there was disagreement in the accuracy, the items were discussed to reach a consensus, utilizing PubMed Central as the source for related scholarship to the given topic. The Blooming Anatomy Tool classifies questions based on whether they require basic knowledge (tier 1), comprehension (tier 2), application (tier 3), or analysis (tier 4) of the information [24]. As such, questions in this study were classified into either “lower order," including questions that fall into tiers 1 and 2, or “higher order," including questions that fall into tiers 3 and 4. To be considered higher order, per the Blooming Anatomy Tool, the questions required the student to infer, predict, judge, critique, or analyze the information given in the question, commonly as part of a clinical vignette. Authors SL and JG reviewed the questions for taxonomy level individually (percent agreement for the total 510 questions = 97.65%). Discrepancies were discussed, using the definition that higher-order questions were required to include the application of clinical knowledge to a patient case to answer the question. Additionally, each question was evaluated for the presence of various technical items noted in the NBME Item-Writing Guide, including (1) negatively structured stems (e.g., except); (2) the presence of a clinical vignette; (3) if the stems were complicated (defined as a stem that lacks focus and requires test takers to do something irrelevant and difficult to answer an item, such as ranking Roman numerals); and (4) the presence of grammatical clues (i.e., if the lead helps test takers eliminate options based on grammatical clues, such as ending the stem with “most likely to be an:”, meaning only options that start with vowels are viable).

Answer choices were also evaluated across seven parameters: (1) similarity among options; (2) complexity of options (e.g., each option is too long); (3) uniformity in length; (4) repetition of words between the question stem and the correct answer; (5) inclusion of “none of the above” or “all of the above” options; (6) presence of absolute terms (e.g., “always” or “never” in the options); and (7) convergence (i.e., when the correct answer has the most in common as other options, such as giving mostly arteries as options, but then including one nerve).

**Figure 1:**
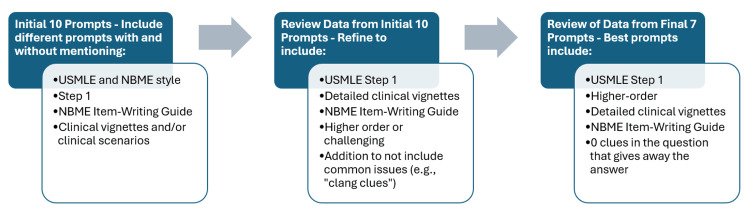
Summary of prompt generation and refinement. Figure 1 was created using Microsoft Word (Microsoft Corporation, Redmond, Washington). USMLE: United States Medical Licensing Examination; NBME: National Board of Medical Examiners

After the initial 10 prompts were evaluated, seven additional prompts were entered and evaluated, each on a new browser, to generate five MCQs across all six systems. The new prompts were developed based on the analysis of the accuracy and quality of the initial 10 prompts, including generating a greater number of “higher order” questions, reducing repeated words in question stems, and including more clinical vignettes. Figure 1 outlines the review process.

The seven new prompts were (1) “Create 5 USMLE Step 1 type, higher order, multiple choice questions using detailed clinical vignettes to apply the gross anatomy and embryology USMLE learning objectives of the ____________ system to clinical scenarios and following the NBME Item Writing Guide.” (2) “Create 5 USMLE Step 1 type, challenging, multiple choice questions using detailed clinical vignettes to apply gross anatomy and embryology USMLE learning objectives of the ____________ system to clinical scenarios and following the NBME Item Writing Guide.” (3) “Create 5 USMLE Step 1 type, challenging, higher order, multiple choice questions using detailed clinical vignettes to apply the gross anatomy and embryology USMLE learning objectives of the ____________ system to clinical scenarios and following the NBME Item Writing Guide.” (4) “Create 5 USMLE Step 1 type, higher order, multiple choice questions using detailed clinical vignettes to apply the gross anatomy and embryology of the ____________ system to clinical scenarios and following the NBME Item Writing Guide. Also ensure there is only a single best answer.” (5) “Create 5 USMLE Step 1 type, higher order, multiple choice questions using detailed clinical vignettes to apply the gross anatomy and embryology of the ____________ system to clinical scenarios and following the NBME Item Writing Guide. Ensure there are no repeated key words in the questions and answers.” (6) “Create 5 USMLE Step 1 type, higher order, multiple choice questions using detailed clinical vignettes to apply the gross anatomy and embryology of the ____________ system to clinical scenarios and following the NBME Item Writing Guide. Ensure there are no ‘clang clues.’” (7) “Create 5 USMLE Step 1 type, higher order, multiple choice questions using detailed clinical vignettes to apply the gross anatomy and embryology of the ____________ system to clinical scenarios and following the NBME Item Writing Guide. Ensure there are no clues in the question that gives away the answer.”

Similarly, each question that ChatGPT produced from the seven new prompts was evaluated against the same five criteria. In total, 510 questions (i.e., 85 questions per organ system) were generated and analyzed using ChatGPT. All data were analyzed in Microsoft Excel (Microsoft Corporation, Redmond, Washington).

## Results

Initial 10 prompts

ChatGPT generated five questions for each system for the initial 10 prompts (n = 50 per system, n = 300 questions total), and the results of the analysis of these questions and answers are presented in Table 2.

**Table 2:** Evaluation of ChatGPT-generated questions from the initial 10 prompts (total questions = 300). ^a^Based on the Blooming Anatomy Tool [24]

​	Accuracy​	Taxonomy (# of higher-order questions)​^a^	Grammatically correct​	Negative in question stem​	Except in question stem​	Includes vignette in question stem​	Complicated question stem​	Grammatical clues ​	Similar options ​	Complex options	Similar option lengths​	Repeated words	None/all of the above	Absolute terms	Convergence
Musculoskeletal​	​45 (90%)	0 (0%)​	48 (96%)​	0 (0%)​	0 (0%)​	37 (74%)​	0 (0%)​	0 (0%)​	50 (100%)	0 (0%)	49 (98%)	8 (16%)	1 (2%)	1 (2%)	2 (4%)
Digestive​	45 (90%)	10 (20%)​	50 (100%)​	1 (2%)​	0 (0%)​	35 (70%)​	0 (0%)​	2 (4%)​	47 (94%)	2 (4%)	49 (98%)	8 (16%)	2 (4%)	1 (2%)	0 (0%)
Nervous​	45 (90%)	2 (4%)​	50 (100%)​	0 (0%)​	0 (0%)​	42 (84%)​	0 (0%)​	0 (0%)​	50 (100%)	0 (0%)	50 (100%)	4 (8%)	0 (0%)	0 (0%)	0 (0%)
Cardiovascular​	33 (66%)	0 (0%)​	50 (100%)​	0 (0%)​	0 (0%)​	19 (38%)​	0 (0%)​	0 (0%)​	49 (98%)	3 (6%)	50 (100%)	6 (12%)	1 (2%)	0 (0%)	1 (2%)
Respiratory​	34 (68%)	11 (22%)​	50 (100%)​	0 (0%)​	0 (0%)​	30 (60%)​	0 (0%)​	0 (0%)​	50 (100%)	0 (0%)	50 (100%)	13 (26%)	0 (0%)	0 (0%)	0 (0%)
Urinary​	38 (76%)	0 (0%)​	50 (100%)​	1 (2%)​	0 (0%)​	26 (52%)​	0 (0%)​	0 (0%)​	50 (100%)	0 (0%)	50 (100%)	6 (12%)	0 (0%)	0 (0%)	0 (0%)
Total​	240 (80%)	23 (7.7%)​	298 (99.3%)​	2 (0.67%)​	0 (0%)​	189 (63%)​	0 (0%)​	2 (0.67%)​	296 (98.7%)	5 (1.7%)	298 (99.3%)	45 (15%)	4 (1.3%)	2 (0.67%)	3 (1%)
Average​	​40 (80%)	​3.83 (7.7%)	​49.67 (99.3%)	​0.33 (0.67%)	​0 (0%)	​31.5 (63%)	0 (0%)	​0.33 (0.67%)	​49.33 (98.7%)	​0.833 (1.7%)	​49.67 (99.3%)	7.5 (15%)	0.67 (1.3%)	0.33 (0.67%)	0.5 (1%)

Question Accuracy

The questions were found to be accurate 80.0% of the time (n = 240), with cardiovascular having the fewest accurate questions (n = 33, 66.0%), while musculoskeletal, digestive, and nervous system questions were equally the most accurate (n = 45, 90.0%). Most of the accuracy issues were due to multiple correct answers, such as “During the process of limb development, which embryonic structure gives rise to the bones and connective tissues of the limb?”, in which both “lateral plate mesoderm” and “somatic mesoderm” were viable answer options.

Question Topic

While many of the questions focused on embryology (n = 230, 76.7%), for many of these, gross anatomy was also relevant to the question stem and needed to be applied to select the correct answer. For example, “A newborn is diagnosed with hydrocephalus, characterized by an enlarged head and increased intracranial pressure. Imaging reveals dilation of the lateral and third ventricles. Which of the following conditions is this most likely associated with during embryonic development?”

Question Stem

Overall, only 23 questions (7.7%) were classified as higher-order questions, but most of the questions (n = 298, 99.3%) were grammatically correct. Question stems were evaluated for whether they contained negatives or “except”, included a vignette, were complicated, and if there were grammatical clues between the question stem and the correct answer choice. The question stems rarely contained negatives (n = 2, 0.67%), and never used the word “except” (n = 0, 0.0%). A majority of questions contained a clinical vignette (n=189, 63.0%). None of the question stems were deemed complicated (n = 0, 0.0%). Grammatical clues between the question stem and the correct answer choice also occurred infrequently (n = 2, 0.67%).

Answer Choices

The answer choices were often similar (n = 296, 98.7%), and rarely complex (n = 5, 1.7%). Repeated words between the question stem and the correct answer choice occurred in 45 questions (n = 45, 15.0%). None/all of the above was present infrequently (n = 4, 1.3%), and absolute terms were similarly uncommon (n = 2, 0.67%). Convergence occurred in 1% of questions (n = 3, 1.0%).

Additional seven prompts

The new questions (n = 35 per system, n = 210 questions total) were evaluated using the same criteria as the initial 10 prompts (Table 3).

**Table 3:** Evaluation of ChatGPT-generated questions from the modified seven prompts (total questions = 210). ^a^Based on the Blooming Anatomy Tool [24]

​	Accuracy​	Taxonomy (# of higher-order questions)​^a^	Grammatically correct​	Negative in question stem​	Except in question stem​	Includes vignette in question stem​	Complicated question stem​	Grammatical clues ​	Similar options ​	Complex options	Similar option lengths​	Repeated words	None/all of the above	Absolute terms	Convergence
Musculoskeletal​	26 (74.3%)	18 (51.4%)	35 (100%)​	0 (0%)​	0 (0%)​	35 (100%)	1 (2.9%)​	0 (0%)​	35 (100%)​	0 (0%)​	35 (100%)​	5 (14.3%)​	0 (0%)​	0 (0%)​	1 (2.9%)​
Digestive​	22 (62.9%)	21 (60%)	35 (100%)​	0 (0%)​	0 (0%)​	35 (100%)	0 (0%)​	0 (0%)​	35 (100%)​	0 (0%)​	35 (100%)​	4 (11.4%)​	0 (0%)​	0 (0%)​	2 (5.7%)​
Nervous​	27 (77.1%)	19 (54.3%)	35 (100%)​	0 (0%)​	0 (0%)​	34 (97.1%)	1 (2.9%)​	0 (0%)​	34 (97.1%)​	0 (0%)​	34 (97.1%)​	4 (11.4%)​	0 (0%)​	0 (0%)​	1 (2.9%)​
Cardiovascular​	30 (85.7%)	16 (45.7%)​	35 (100%)​	0 (0%)​	0 (0%)​	35 (100%)	0 (0%)​	2 (5.7%)​	35 (100%)​	0 (0%)​	35 (100%)​	4 (11.4%)​	0 (0%)​	0 (0%)​	0 (0%)​
Respiratory​	21 (60%)	11 (31.4%)​	35 (100%)​	0 (0%)​	0 (0%)​	34 (97.1%)	0 (0%)​	0 (0%)​	35 (100%)​	0 (0%)​	35 (100%)​	1 (2.9%)​	0 (0%)​	0 (0%)​	0 (0%)​
Urinary​	22 (62.9%)	1 (2.9%)​	35 (100%)​	0 (0%)​	0 (0%)​	26 (74.3%)	1 (2.9%)​	0 (0%)​	35 (100%)​	1 (2.9%)​	35 (100%)​	15 (42.9%)​	0 (0%)​	0 (0%)​	0 (0%)​
Total​	148 (70.5%)	86 (41%)​	210 (100%)​	0 (0%)​	0 (0%)​	199 (94.8%)	3 (1.4%)​	2 (1%)​	209 (99.5%)​	1 (0.5%)​	209 (99.5%)​	33 (15.7%)​	0 (0%)​	0 (0%)​	4 (1.9%)​
Average​	24.67 (70.5%)	14.33 (41%)	35 (100%)	​0 (0%)	0 (0%)	​33.17 (94.8%)	​0.5 (1.4%)	​0.33 (1%)	​34.83 (99.5%)	​0.17 (0.5%)	​34.83 (99.5%)	5.5 (15.7%)	0 (0%)	0 (0%)	0.67 (1.9%)

Question Accuracy

**Figure 2:**
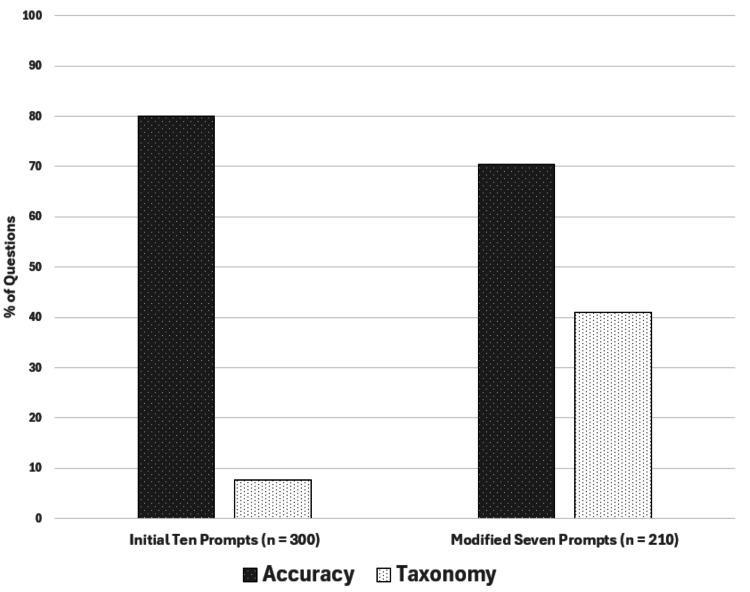
Comparison of accuracy and taxonomy levels (percentage of higher-order questions) between the initial 10 prompts and the modified seven prompts. Figure 2 was created using Microsoft Excel (Microsoft Corporation, Redmond, Washington).

The overall accuracy of the questions for the new prompts decreased to 70.5% (n = 148), which was also due to some of the questions having multiple correct answers. Interestingly, cardiovascular had the highest number of accurate questions (n = 30, 85.7%), while respiratory had the lowest number of accurate questions (n = 21, 60.0%). Figure 2 highlights the differences in question accuracy and taxonomy between the initial 10 prompts and the modified seven prompts.

Question Topic

Similar to the initial 10 prompts, most questions focused on embryology content (n = 150, 71.4%), though gross anatomy was relevant for some of these embryology prompts, such as “A 7-year-old boy presents with episodes of exertional dyspnea and fatigue. Cardiac auscultation reveals a diastolic murmur at the left lower sternal border. An echocardiogram shows a defect between the left and right ventricles. Which developmental process is likely disrupted, resulting in this condition?”

Question Stem

The percentage of higher-order questions increased to 41.0% (n = 86), and all 210 (100.0%) were grammatically correct. The percentage of questions with a clinical vignette in the question stem increased from 63.0% to 94.8%. Notably, despite prompting ChatGPT to write questions according to the NBME writing guide, the occurrence of repeated words in the question stem and correct answer choice did not drastically change with the modified prompts (15.0% of initial questions and 15.7% of new questions).

Several parameters related to the question stems and answer choices remained largely unchanged or showed only slight variation following the use of the modified prompts. The presence of negatives in the question stem decreased to 0 (n = 0, 0.0%), and the inclusion of “except” in the question stem remained consistently absent (n = 0, 0.0%). The proportion of complicated question stems increased slightly to 1.4% (n = 3). The grammatical clues in the question stem increased to 1.0% (n = 2).

Answer Choices

Similar answer choices were found in 99.5% of questions (n = 209), and complex answer choices were rare (n = 1, 0.5%). Uniformity in option lengths persisted with the modified prompts (n = 209, 99.5%). Absolute terms and “none/all of the above” were found in none of the answer options with the modified prompts (n = 0, 0.0%), and convergence was found in 1.9% of the answer choices (n = 4).

## Discussion

The primary goal of this study was to evaluate the quality of ChatGPT-generated gross anatomy and embryology MCQs by exploring different prompts without providing specific curricular content or learning objectives. The findings indicate that the questions performed well in specific areas, including grammatical accuracy, avoiding negative phrasing or “except” in the question stem, and maintaining clear, concise question stems. Regarding answer choices, the questions generally featured simple and comparable options, minimized unnecessary complexity, and excluded “none/all of the above” responses. However, areas for improvement included a tendency toward lower-order questions and answer choices that often repeated words, which could inadvertently provide clues to test takers.

These findings are consistent with prior studies analyzing ChatGPT’s question generation capabilities. Zuckerman et al. [25] found that while ChatGPT performed well in generating lower-order questions, more complex items often required additional editing by faculty to meet educational standards. Similarly, in a review of studies examining ChatGPT’s ability to generate MCQs, prompts that did not reference an established format produced lower-quality questions, while case-based questions tended to perform better [26]. Although our prompts explicitly requested the inclusion of clinical vignettes, the overall accuracy of the questions generated remained variable, suggesting that prompt structure alone is insufficient to guarantee question quality.

Modifying prompts successfully increased the proportion of questions containing clinical vignettes and the proportion of higher-order questions. This study found that prompts using "USMLE-style" phrasing generated more higher-order questions with longer, more detailed vignettes compared to those using "NBME-style" phrasing. However, prompts designed to generate more clinically complex questions came at the expense of accuracy, suggesting a tradeoff between complexity and factual reliability in AI-generated content. In developing an optimal prompt, higher-quality questions resulted from those that were longer, more specific, and used precise language. Incorporating terms such as "avoid clues" and explicit references to the NBME Item-Writing Guide improved output quality, consistent with others who similarly emphasize that effective question generation requires clearly defined objectives, a specific format, and the inclusion of complex medical terminology while minimizing ambiguity [27,28].

While most accuracy issues were associated with multiple correct answers, some questions were determined to be incorrect for other reasons. One reason was that the question prompts did not properly align with the answer options to be fully correct and, therefore, counted as incorrect. For example, the question "During embryonic development, which structure eventually forms the right atrium?" listed "sinus venosus" as the correct answer; however, this is only partially correct, as the sinus venosus gives rise solely to the smooth-walled portion of the right atrium. In other instances, the question and options were entirely incorrect. One example includes questions regarding the forebrain in the nervous system, which includes structures associated with the primary brain vesicle known as the prosencephalon that gives rise to both the telencephalon and the diencephalon. Some questions asked which primary vesicle the forebrain is derived from and noted the telencephalon as the correct answer. Another point to note was that, while the prompts focused on embryology and gross anatomy, some histology questions were also included on glial cells in the nervous system (n = 5) and the layers of the gastrointestinal tract (n =2). For the purposes of this study, these questions were considered “gross anatomy” as they did not focus on development.

The challenge in prompting ChatGPT to generate complex, accurate, application-based questions likely reflects the nature of its training. ChatGPT was trained on a wide variety of openly sourced information and optimized to generate human-like responses through reinforcement learning with human feedback [29], identifying and using word patterns to construct contextually plausible output [30]. While this enables ChatGPT to produce well-constructed responses, it does not guarantee factual accuracy. This is further compounded by the well-documented phenomenon of AI hallucination, in which language models generate output that is entirely fabricated yet sounds plausible [31]. This study expands on previous research where accuracy issues have been seen when utilizing LLMs in question generation and exam performance [17,18,32]. A representative example of pattern-based generation was observed in questions related to the gastrointestinal system, where 8 of the generated questions used the phrase "double bubble sign" to refer to duodenal atresia - a commonly referenced association in medical education resources. While this demonstrates ChatGPT's ability to identify and reproduce high-yield clinical associations, it also highlights its tendency toward recall-based question generation rather than the higher-order clinical reasoning required by NBME-style examinations. Based on these limitations, this study suggests that ChatGPT performs better at generating lower-order questions that assess recall of common clinical associations than higher-order questions requiring synthesis and application of clinical knowledge.

Despite these limitations, ChatGPT offers a meaningful advantage in speed of question generation. When ChatGPT-generated questions are subsequently reviewed and edited by faculty, overall efficiency in question development improves even when accounting for the editing process [25]. Questions should therefore be reviewed against NBME standards prior to use in any formal assessment, with particular attention to removing answer choice clues and ensuring higher-order cognitive demand.

Limitations

This study has several limitations that should be considered when interpreting its findings. First, the total number of questions generated limits the generalizability of the results, and a larger question set across a broader range of topics would provide a more comprehensive evaluation of ChatGPT's capabilities. Second, the classification of questions as higher- or lower-order is inherently subjective; while this study defined higher-order questions as those requiring application of clinical knowledge to a patient case, this distinction may be interpreted differently across evaluators and institutions, and reasonable disagreement exists regarding question classification. To address this limitation, the research team discussed the questions when there were discrepancies between evaluators in their designations in order to reach a consensus. Third, the scope of prompt types explored, while systematic, does not exhaustively represent all possible approaches to prompt design, and different phrasing strategies beyond those tested here may yield meaningfully different results.

Additionally, there was no inferential statistical testing done as part of this study looking at learner psychometrics. Previous research has highlighted the variability of MCQs used in medical education in regard to their psychometrics, including difficulty and discrimination [33]. As such, there would be additional value in looking at these metrics when reviewing questions generated using LLMs. Finally, as LLMs are continuously evolving, the findings of this study reflect the capabilities of the 4.0 version of ChatGPT used at the time of data collection and may not be representative of current or future model performance as updates become available.

Future directions

Future studies should aim to identify the specific keywords and prompt structures that most reliably generate higher-order, clinically complex questions reflective of NBME examination standards, including questions that require synthesis of knowledge across multiple organ systems. Research should also explore whether providing structured prompt templates or prompt refinement strategies can improve both the accuracy and cognitive complexity of AI-generated questions without sacrificing one for the other. Evaluating the performance of newer and more advanced language model versions on the same prompts used in this study would allow for direct longitudinal comparison of model capabilities over time. Additionally, future work should examine whether faculty-edited AI-generated questions perform comparably to entirely human-written questions in formal assessments and whether students can reliably detect inaccuracies in AI-generated content when using it independently as a self-study tool. As LLMs continue to evolve, their ability to generate clinically relevant, higher-order questions may improve, and ongoing evaluation against established educational standards will be essential to determining their appropriate role in medical education item writing.

## Conclusions

In conclusion, the data in this study suggest that ChatGPT performs well in generating lower-order questions that assess foundational knowledge but is less effective at crafting higher-order questions that require applying and synthesizing clinical knowledge in complex patient scenarios. Additionally, question quality, as described in the NBME’s Item-Writing Guide, is variable and still requires additional review before use. As such, ChatGPT can still serve as a supplemental tool for medical student question generation, but the accuracy and representativeness of board-style questions will be variable and cannot be assumed. Similarly, ChatGPT has the potential to increase faculty efficiency by streamlining question writing, provided the questions are reviewed prior to use.
